# Intraoperative Monitoring of the Cochlear Nerve during Neurofibromatosis Type-2 Vestibular Schwannoma Surgery and Description of a “Test Intracochlear Electrode”

**DOI:** 10.1055/s-0038-1673649

**Published:** 2019-02-04

**Authors:** Anand V. Kasbekar, Yu Chuen Tam, Robert P. Carlyon, John M. Deeks, Neil Donnelly, James Tysome, Richard Mannion, Patrick R. Axon

**Affiliations:** 1 Cambridge Skull Base Unit, Cambridge University Hospitals NHS Foundation Trust, Cambridge, United Kingdom; 2 Department of Otorhinolaryngology, Head and Neck Surgery, Nottingham University Hospitals NHS Trust, Nottingham, United Kingdom; 3 NIHR Nottingham Biomedical Research Centre, University of Nottingham, Nottingham, United Kingdom; 4 Emmeline Centre, Cambridge University Hospitals NHS Foundation Trust, Cambridge, United Kingdom; 5 Medical Research Council Cognition and Brain Sciences Unit, Cambridge, United Kingdom

**Keywords:** cochlear nerve monitoring, neurofibromatosis type 2, hearing preservation, EABR

## Abstract

**Objectives** A decision on whether to insert a cochlear implant can be made in neurofibromatosis 2 (NF2) if there is objective evidence of cochlear nerve (CN) function post vestibular schwannoma (VS) excision. We aimed to develop intraoperative CN monitoring to help in this decision.

**Design** We describe the intraoperative monitoring of a patient with NF2 and our stimulating and recording set up. A novel test electrode is used to stimulate the CN electrically.

**Setting** This study was set at a tertiary referral center for skull base pathology.

**Main outcome measure** Preserved auditory brainstem responses leading to cochlear implantation.

**Results** Electrical auditory brainstem response (EABR) waveforms will be displayed from different stages of the operation. A cochlear implant was inserted at the same sitting based on the EABR.

**Conclusion** Electrically evoked CN monitoring can provide objective evidence of CN function after VS excision and aid in the decision-making process of hearing rehabilitation in patients who will be rendered deaf.

Patients with neurofibromatosis type 2 (NF2) report that their greatest problem is deafness. 1 If a patient is due to be rendered bilaterally deaf as is eventually the case in NF2 then the choice of hearing rehabilitation lies between a cochlear implant and an auditory brainstem implant (ABI). It is a usual practice to use an ABI in the NF2 population as preserving the cochlear nerve during NF2-related vestibular schwannoma (VS) removal is difficult, and anatomical preservation does not guarantee functional preservation. 2

Several centers around the world have inserted a cochlear implant following objective cochlear nerve testing at the time of VS surgery for hearing rehabilitation. 3 4 5 6 This is especially relevant when the approach to the tumor is the translabyrinthine route which will destroy any residual hearing. In patients who will eventually lose their hearing bilaterally such as in NF2, it is essential that the best means of hearing rehabilitation is provided. Compared with the conventional ABI used in such cases, the cochlear implant provides better hearing. 7 Inserting a cochlear implant at the same sitting as the VS removal will reduce the number of operations and procedures that such patients will need to undertake. Implantation as soon as is possible is also preferred as it reduces the chances of cochlear ossification preventing cochlear implantation. 5 8

## Methods of Monitoring the Cochlear Nerve

**Fig. 1:**
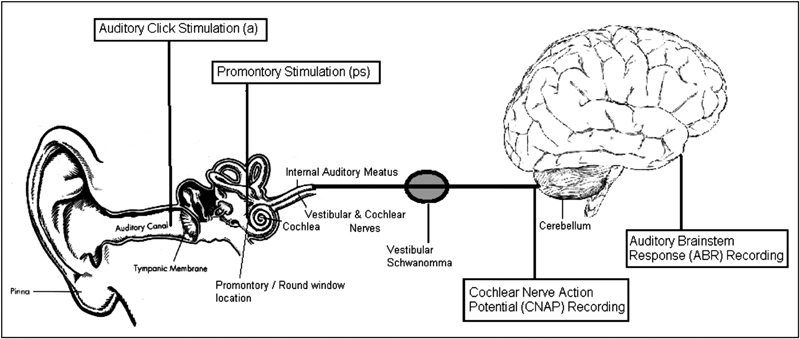
Simplified schematic diagram of the auditory pathway. The boxed text indicates possible sites for stimulation and recording along the auditory pathway during vestibular schwannoma surgery.

Surgeons and neurophysiologists have tried various systems to monitor the cochlear nerve to predict its function postoperatively. 4 9 10 11 12 These are based on the principle that the nerve is stimulated at or near the cochlea and this stimulus is detected either by measuring the electrical activity that is induced directly from the cochlear nerve (cochlear nerve action potential [CNAP]) or further along the auditory pathway (the auditory brainstem response [ABR]) (see Fig. 1 ).

The stimulus can be in the form of auditory “clicks” introduced into the ear canal via earphones (ABR) or via electrical stimulation of the cochlea and the cochlear nerve resulting in an electrical auditory brainstem response (EABR). The most investigated methods of intraoperative cochlear nerve monitoring have been ABR and auditory click-stimulated CNAP. 13 14 They are also the methods of choice that are being utilized in most skull base centers that operate on VS.

Any intraoperative monitoring that utilizes ABR techniques is at a disadvantage as it does not allow real-time feedback of cochlear nerve function. 15 CNAP is preferable as it eliminates the need for recording many hundreds of repetitions to obtain a reliable waveform, as the CNAP amplitude is much larger than that of an ABR. There is also less background electrical noise when recording from the nerve directly than from scalp electrodes. 15

## Changes in the Monitoring Waveforms: What Is Significant?

Deciding when the ABR or CNAP is indicative of cochlear nerve damage is not straightforward. During the ABR, the decrease in amplitude or the increase in latency of the waves are the two means of determining when there might be damage to the nerve. 16 The main waves looked at during an ABR are waves I, III, and V, with wave V being the most robust. Wave I, III, and V latencies, as well as interwave latencies of waves I–III, I–V, and III–V, are also commonly analyzed. 17 Monitoring with CNAP usually requires an evaluation of amplitude change and action potential morphology. The process can be quite subjective when trying to determine if a waveform exists within a background of the electrical artifact.

Many clinicians support an arbitrary ABR warning criterion of a 50% decrease in amplitude and/or a 1 ms increase in latency for wave V. 18 19 This is not an unreasonable criterion, but it has not been demonstrated to be predictive of postoperative function for monitoring of the auditory system. 20 It is also likely that the optimal warning criteria depend on the type of surgical procedure. For CPA surgeries other than tumor resection, such as neurovascular decompression, hearing loss occurs only when there is a permanent loss of wave V. 21 22

## Monitoring during Translabyrinthine Surgery

Acoustic stimuli, however, cannot be undertaken following translabyrinthine surgery to gain access to the VS as residual hearing is lost during the drilling of the vestibular apparatus with loss of inner ear fluid. Any monitoring employed during a translabyrinthine surgical approach must, therefore, utilize electrical stimulation of the cochlear nerve. This can be via stimulating at the middle ear promontory or round window niche with ABR (far-field) or CNAP (near-field) recording. Lloyd et al 6 recently reported their use of electrically stimulated CNAP to gage the integrity of the cochlear nerve before cochlear implantation for hearing rehabilitation following translabyrinthine resection of VS.

This article describes one of our first attempts at intraoperative EABR in an NF2 patient to insert a cochlear implant to aid in hearing rehabilitation. We utilized the techniques we had learned over the last 5 years to monitor the cochlear nerve so we could achieve our aim of optimum hearing rehabilitation.

## Methods

A 37-year-old female patient affected by NF2, in December 2013 had undergone a left-sided translabyrinthine excision of VS along with insertion of a sleeper ABI. This procedure had removed all hearing on his left side, but the patient had serviceable hearing on the right side. During 2014, the right-sided hearing levels deteriorated significantly to the American Academy of Otolaryngology and Head Neck Surgery (AAOHNS) Class D 23 due to a slowly increasing 2 cm VS. The tumor was mainly within the CPA with some extension into the fundus of the internal auditory meatus (IAM). The decision to remove the tumor with an attempt to preserve the cochlear nerve was taken functionally. This would allow a cochlear implant to be inserted with better hearing rehabilitation.

**Fig. 2:**
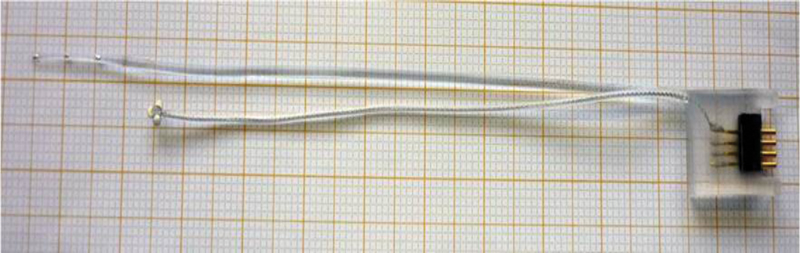
Stimulating intracochlear electrode array manufactured by MED-EL (Innsbruck, Austria).

The following monitoring techniques were developed as part of a research study registered with the Cambridgeshire Research Ethics Committee (reference 08/H0308/76). The Medelec Synergy system (VIASYS HealthCare UK, Surrey, United Kingdom) was used to monitor the cochlear nerve via EABR (using far-field electrodes). Our previous work (unpublished) showed an intracochlear electrode to be superior in electrically stimulating the auditory pathway. We were also unable to use our custom-made electrode due to problems with manufacturing the device. In response to our request, a cochlear implant manufacturing company MED-EL (Innsbruck, Austria) was able to provide us with a “test” intracochlear electrode. The test electrode was used as a custom-made device on a named patient basis. This was similar to a cochlear implant electrode array but with four intracochlear electrodes spaced 4.2 mm apart (total array length: 18 mm), and an extracochlear return electrode if needed which is embedded into the temporalis muscle (see Fig. 2 ). The diameter at the array tip is 0.5 mm.

For monitoring to take place using an intracochlear electrode, access to the middle ear is required, and the electrode is inserted through the round window, similar to cochlear implant surgery. For this case, the entire array was inserted into the cochlea and was stimulated at electrode 2 with the return chosen as electrode 4. The electrode is kept in place with some bone wax securing it near the round window. The time taken for this added surgical step is in our experience not more than a couple of minutes. However, obtaining a baseline waveform from which to begin monitoring can take a few more minutes as stimulation and recording parameters need to be adjusted according to the patient and surgical factors. For example, a higher current stimulus is needed if high electrode impedance is encountered. It is important to note that this technique will likely destroy any residual hearing and therefore is usually employed in cases of the translabyrinthine approach when the hearing is sacrificed. This technique is not suitable for stimulation via broadband “clicks” to monitor residual hearing in which only recording electrodes are needed.

The MED-EL electrode is stimulated by an ABI stimulator box, which generates biphasic stimulation pulses and is controlled by the MED-EL diagnostic interface box via the coil placed on the ABI stimulator box. The stimulation pulses are synchronized with the recording system using a standard “TTL” signal. Table 1 details the specific stimulation method and the parameters used. Testing was undertaken at various stages during surgery and at the end of tumor removal.

**Table 1:** Displaying the stimulation and recording parameters for cochlear implant EABR

Parameter	
Stimulus presentation	Med-el intracochlear electrode 2
Return electrode	Intracochlear electrode 4
Type of stimulus	Electrical
Rate of stimulation	31/s
Level of stimulation	Variable
Pulses	Biphasic, variable phase width 50–200 μs
Recording Montage (surface electrodes)	Positive: high forehead (Fz) Negative: C7 Ground: contralateral shoulder
Filter	
High pass	30 Hz
Low pass	3,000 Hz
Amplifier gain	50,000
Sweep time	10 ms
Number of sweeps	2,048

Abbreviation: C7, electrode placement over the seventh cervical vertebrae.

Initial wave III and V amplitude and latency were measured as a baseline at the prelabyrinthectomy stage. Criteria to warn the surgeon as to impending cochlear nerve damage was a wave V latency prolongation of over 1 ms and amplitude reduction greater than 50%.

## Results and Analysis

Initial testing prelabyrinthectomy determined that 1,000 cochlear units were an adequate stimulation to produce an EABR. A selection of recordings will be displayed at various stages along the surgery along with latency values where appropriate.

**Fig. 3:**
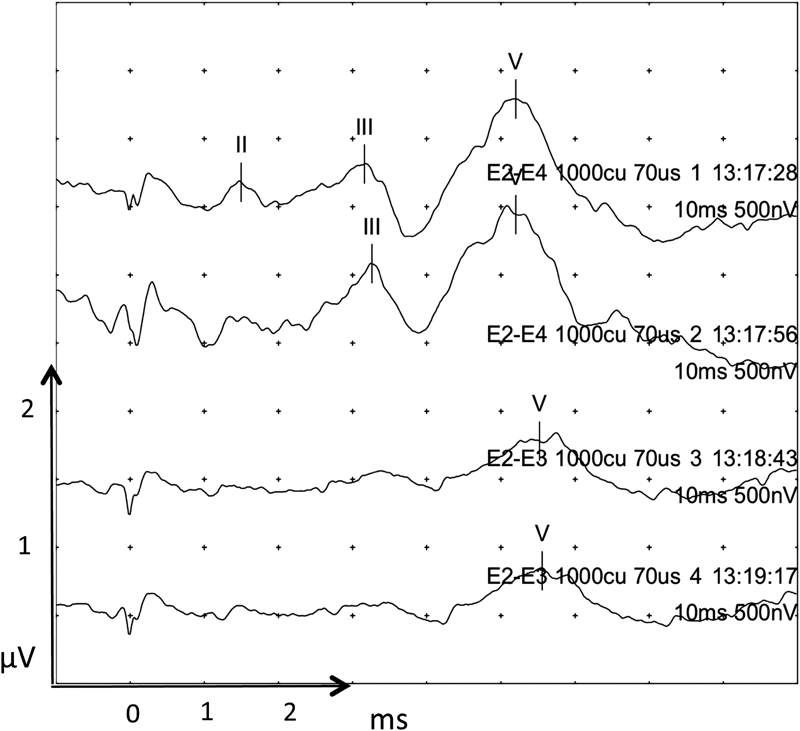
Prelabyrinthectomy EABR. The bottom two traces utilized a bipolar stimulation method with stimulation at electrode 2 and return electrode 3. This stimulation technique produced a poorer waveform and was therefore not utilized further. Waves are marked where seen. Wave I where marked may be stimulation artifact. Individual traces are labeled with the stimulating and return electrode number (E), cochlear unit (CU) level, μs phase width, and time of recording. Each division/dot in the x-axis is 1 ms, and 0.5 μV in the y-axis. EABR, electrical auditory brainstem response.

**Fig. 4:**
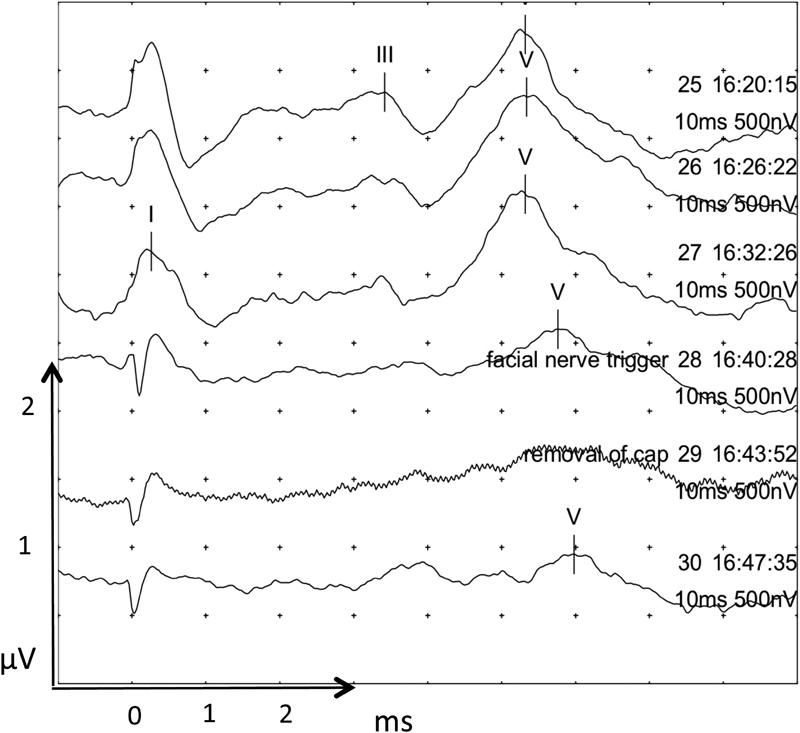
Further tumor and nerve dissection EABR (traces 25 to 27). Loss of a clear wave III and widening of wave V morphology was seen with an increase in wave V latency to 5.3 ms. Traces 28 to 30 were undertaken as the tumor capsule was being dissected from the cochlear nerve causing a >50% decrease in wave V amplitude and an increase in latency of 0.6 to 0.9 ms. Wave I where marked may be stimulation artifact. EABR, electrical auditory brainstem response.

The top two traces in Fig. 3 showed a lengthening of EABR before wave III indicating pathology proximal to the cochlear nucleus. Postlabyrinthectomy the EABR was intact as would be expected. During and after IAM drilling the EABR was still clearly recognizable, without loss of wave III and V amplitude or latency. During tumor and nerve dissection, the EABR was preserved for a while till further tumor and nerve dissection ( Fig. 4 , traces 25–27) caused loss of a clear wave III and widening of wave V morphology with an increase of wave V latency to 5.3 ms. Traces 28 to 30 were undertaken as the tumor capsule was being dissected from the cochlear nerve causing a >50% decrease in wave V amplitude and an increase in latency of 0.6 to 0.9 ms.

**Fig. 5:**
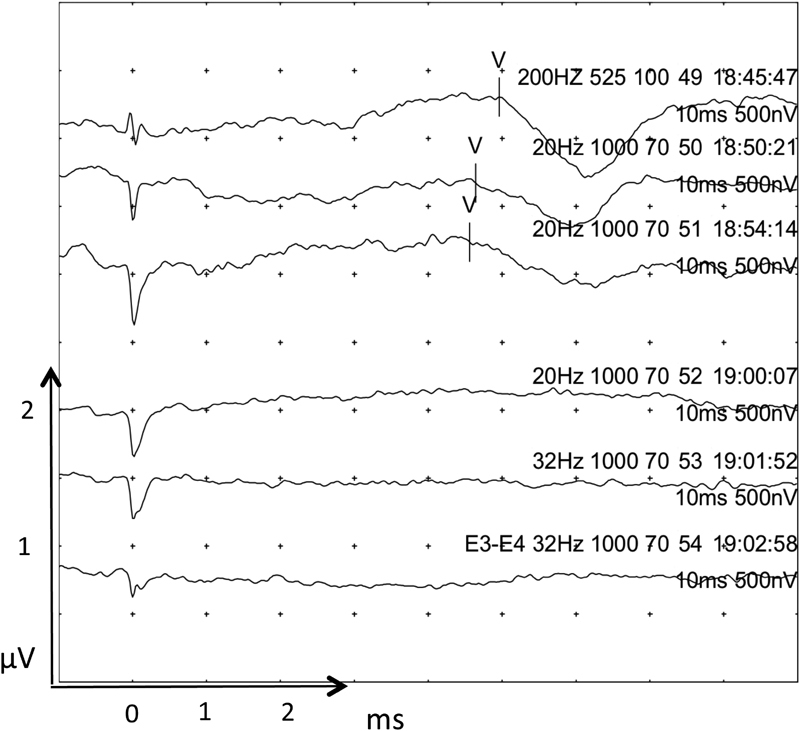
During further tumor and nerve dissection EABR. Recordings 49 to 51 showed a possible wave V with grossly widened morphology and >50% amplitude reduction. Recordings 52 to 54 show a complete loss of wave V with further tumor dissection from the cochlear nerve. Waves are marked where seen. Wave I where marked may be stimulation artifact.

At this stage in operation, the surgeon was warned of the possible damage to the cochlear nerve when a >50% decrease in wave V amplitude was detected. At the same time, the facial nerve was depolarized and detected by the facial nerve monitoring system. The surgeon stopped tumor dissection and allowed the nerve to recover for a few minutes. Recovery of baseline EABR did not occur, and therefore the surgeon continued gentle dissection changing his dissection technique and approach. During further tumor and nerve dissection ( Fig. 5 , recordings 49–51), the EABR showed a possible wave V with grossly widened morphology and >50% amplitude reduction. Recordings 52 to 54 show a complete loss of wave V with further tumor dissection from the cochlear nerve.

**Fig. 6:**
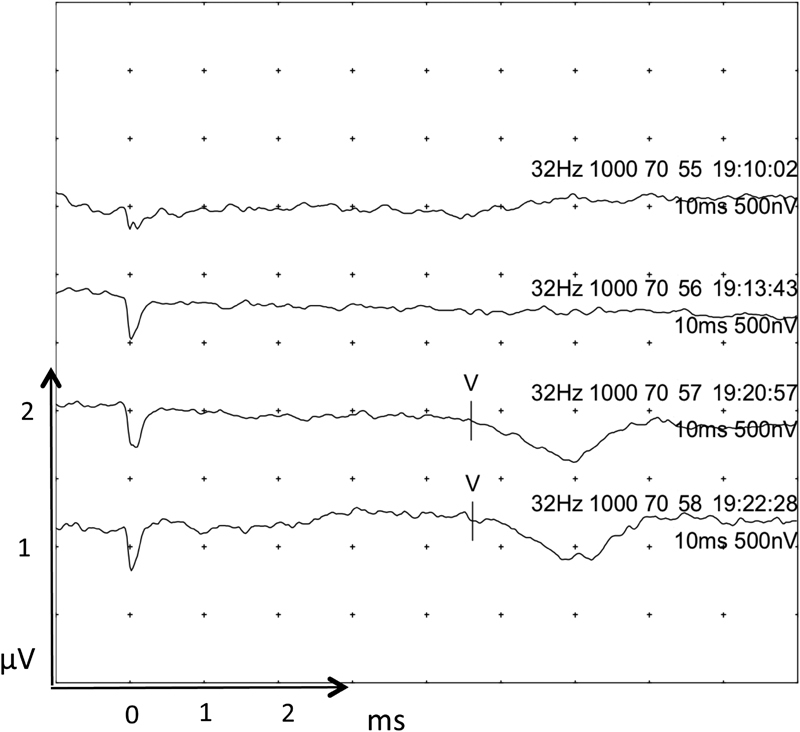
Completion of tumor resection EABR. Recordings 55 and 56 showed a complete loss of EABR. Traces 57 and 58 are recordings after the addition of the antispasmodic agent papaverine applied over the cochlear nerve. They display some features of a wave V. Waves are marked where seen.

At the completion of tumor resection EABR there was a complete loss of EABR ( Fig. 6 , recordings 55–56). At this point, the cochlear nerve was bathed in the antispasmodic agent papaverine. This improved the EABR and showed features of wave V with a characteristic downward slope. This is easier to appreciate when compared with recordings 49 to 51 in Fig. 5 .

At this stage, a Nucleus Freedom cochlear implant was inserted into the same side as tumor removal had taken place. Further cochlear implant EABR testing did not produce convincing EABR traces. Increasing the stimulation level eventually caused the facial nerve to depolarize at which point testing was stopped.

## Discussion

Our aim at the start of the operation was to preserve the cochlear nerve leading to cochlear implantation functionally. This should however not override the primary objective, which was total/near-total tumor removal with preservation of the facial nerve. Initially, monitoring must take place with far-field techniques using EABR, but once access is gained to the proximal part of the cochlear nerve, a direct cochlear nerve recording electrode, such as the Cueva electrode (AD-TECH Medical Instrument Corporation) can be attached allowing CNAP to take place. 10 Unfortunately, CNAP monitoring, in this case, was not possible as it was problematic to place the Cueva electrode in a suitable position to record reliable waveforms. The potential problems with recording CNAP with electrical stimulation, rather than the usual auditory stimulation are numerous and include electrical artifact and current shunting due to cerebrospinal fluid flow. 11 Our monitoring, therefore, consisted of EABR throughout.

Changes in EABR waveform vary between near-field (direct cochlear nerve recording, e.g., CNAP) and the far-field (using scalp electrodes) technique. The former will allow near-real-time monitoring. Far-field techniques rely on averaging several hundred responses to obtain a reliable waveform as was utilized in our case. This method introduces a delay between a damaging surgical maneuver and an abnormal waveform of between 15 seconds and 1 minute. The electrophysiologist must then interpret the waveform changes from the baseline waveform and warn the surgeon. In practice, this takes a few seconds. Software packages that can detect changes in EABR waveform are not in widespread use due to the complexity of the EABR and the pattern changes that can result due to cochlear nerve damage. We used a highly experienced electrophysiologist to interpret the EABR waveform changes during the operation visually.

### Effects on the EABR during Surgery

During intraoperative monitoring of the cochlear nerve, there are obvious surgical maneuvers that may change the auditory-evoked potentials. Systemic factors, such as hypotension or hypothermia, during surgery, can affect the waveform amplitude and latency. Irrigation of the surgical field will cause a local hypothermic effect and can increase the latency of wave I and interwave latencies. 24 This effect is greater when temperatures are less than 26°C and can double latency times normally measured at 37°C. Waveform amplitude has a more complex relationship with temperature and can initially increase before decreasing at temperatures below 30°C. 25 Effects of anesthesia have less of an effect on auditory-evoked potentials. 26 The changes described are usually reversible and therefore irrigation with warm saline or the infusion of intravenous fluids to correct hypothermia and hypotension, respectively, will correct any shift in wave latency and amplitude. The recognition of these abnormalities when there is a shift in waveform latency or amplitude is important so as not to mistake them for cochlear nerve damage.

### A Closer Analysis of the Intraoperative EABR

Initial EABR traces pretumor dissection show wave V latency at 5.1 ms and amplitude at between 0.85 and 1.05 V. This latency value is delayed by almost three standard deviations and approximately 1 ms when compared with the normative EABR data. 27 This increase in latency occurs between waves I and III as the interwave latency wave III to V is normal. This would correlate with a lesion located on the cochlear nerve, which would affect nerve conduction between sites responsible for the generation of waves I and III of the ABR. Drilling of the IAM is commonly associated with sudden loss of auditory-evoked potentials. 28 This, fortunately, did not occur in our case. The mechanism of injury is proposed to be a combination of noise, physical damage, and vascular compromise. 29

An EABR was well preserved until tumor capsule dissection from the cochlear nerve at which point only wave V could be recognized. Traces 28 to 30 ( Fig. 4 ) were undertaken as the tumor capsule was being dissected from the cochlear nerve and shows a >50% decrease in wave V amplitude and an increase in latency from 5.1 to 5.7 ms. This amplitude drop of approximately 50% from baseline was the point at which the surgeon was warned of possible cochlear nerve damage. The EABR, however, did not return despite waiting for 5 minutes. Before the sudden decrease in wave V amplitude, the morphology of wave V was widened (see recordings 25–27 in Fig. 4 ), and this may have been a more sensitive indicator of impending cochlear nerve damage. Measuring the angle between the ascending and descending limb of wave V may provide a means to quantify this abnormal morphology.

Dissecting the tumor from the cochlear nerve is a delicate process and damage to the nerve can occur from mechanical trauma, thermal damage (from electrocautery), or ischemia (vessel damage). Traction injury on the nerve can occur and is most likely to cause harm to the distal end, with avulsion of fragile nerve endings at the cochlea. This would cause a loss of wave I and subsequent waves. It is consequently recommended that should traction be required during dissection; it should be applied toward the cochlea rather than away from it. 30

NF2 tumors tend to be multilobular and often demonstrate a more invasive growth pattern than solitary tumors. 31 Thus, they are harder to separate from the cochlear nerve during dissection, and consequently, there are higher rates of cochlear nerve damage during surgery.

Figs. 5 and 6 display recordings at near completion of tumor dissection and the end of the procedure, respectively. The possible wave V demonstrated latencies, which decreased to approximately 4.6 ms. This is a decrease from the baseline of 5.1 ms. This could have been due to the release of compression of the cochlear nerve from the VS allowing a more normal signal conduction. Another possibility is that cerebellar retraction was released. This would release pressure on the small vessels supplying the cochlear nerve (and reverse any nerve ischemia), or mechanical stretching of the cochlear nerve. 32

### The Use of Papaverine

The addition of the antispasmodic agent papaverine into the surgical field caused a return of wave V, albeit of low amplitude and abnormal morphology. The characteristic negative peak at 5 ms corresponded to the wave V negative peak earlier in the recordings. Papaverine has been used by others in the past with some success in the reversal of neurophysiological changes in the cochlear nerve. 26 33 Papaverine is a phosphodiesterase inhibitor that relaxes the smooth muscle of the vascular system and is routinely used in vascular surgery. 34 The internal auditory artery (or labyrinthine artery) runs within the IAM supplying the inner ear and cochlear nerve and is susceptible to injury, either vasospasm or transection. 26 The addition of papaverine caused a return of wave V and suggested that the blood supply to the cochlear nerve was in spasm and not irreversibly damaged at this time.

The administration of intraoperative steroids is routine in our unit during VS surgery. Steroids have been shown to have a beneficial effect on sudden sensorineural hearing loss. 35 They are also shown to improve cochlear spiral ganglion cell survival due to cochlear nerve compression in the rat model. 36 More recently, therapy with vasoactive treatments perioperatively have shown mixed results in improving the rates of hearing preservation surgery. 37 38 39 Gouveris et al 38 did not find any benefit in administering steroids and vasoactive treatment to patients with the immediate postoperative hearing loss after VS surgery. Unfortunately, no intraoperative ABR traces were obtained, and therefore the patients could not be classified into those that may benefit from such treatment, and those that are unlikely to. Bischoff et al 39 found that only patients with reversible loss of wave V benefited from vasoactive treatment. Furthermore, patients benefited more so when the treatment was initiated preoperatively. 40 Treatments in the studies were also almost universally administered systemically, either intravenously or orally. Our patient did not receive preoperative vasoactive treatment, or the agent systemically. This may have reduced the effect of the papaverine. It was nevertheless interesting to see an abnormal wave V returns with its topical application, lending credibility to its perioperative use in the future.

### The Decision to Insert a Cochlear Implant

With the presence of a wave V, albeit with abnormal morphology, latency, and amplitude according to the American Society of Neurophysiological Monitoring (ASNM) statement, 16 the decision to insert a cochlear implant was taken. This was a complex decision-making process as the choice for the surgeon was either to leave tumor behind to increase the chances of a functioning cochlear nerve, or to remove all the tumor and risk damaging the nerve. Complete tumor removal had taken place in this case, and with the slender hope of a functioning nerve, a cochlear implant was inserted. Unfortunately, no recognizable waveforms could be made out during cochlear implant EABR (not shown). The patient's cochlear implant did not produce any auditory sensation at 3 months postsurgery. At the time of surgery a hybrid ABI–cochlear implant was not available to us, which would have been preferable to implant given the abnormal ABR post excision.

There are however reports of successful outcomes with cochlear implantation despite a lack of an EABR. These cases are, however, in children with auditory neuropathy with a variety of disorders affecting sound processing and not in patients having had surgery affecting the cochlea and cochlear nerve.

Finally, it is worth considering the possible cochlear trauma of using a temporary intracochlear electrode for monitoring purposes. The insertion of such an electrode should be as careful as introducing a real implant electrode, with adequate fixation at the round window to prevent movement during surgery.

## Conclusion

Intraoperative monitoring of the cochlear nerve is especially difficult with electrical stimulation during the translabyrinthine approach. This case demonstrates the problems in interpreting the EABR, and also the challenges in deciding on whether to insert a cochlear implant. The use of the stimulating MED-EL electrode greatly helps in this technique and is invaluable in this setting. The return of an abnormal EABR after its complete loss is not predictive of cochlear nerve functional preservation and does not warrant cochlear implantation. Larger scale studies are required in patients with and without NF2 to corroborate our findings.
